# Metformin represses bladder cancer progression by inhibiting stem cell repopulation via COX2/PGE2/STAT3 axis

**DOI:** 10.18632/oncotarget.8595

**Published:** 2016-04-05

**Authors:** Qiuli Liu, Wenqiang Yuan, Dali Tong, Gaolei Liu, Weihua Lan, Dianzheng Zhang, Hualiang Xiao, Yao Zhang, Zaoming Huang, Junjie Yang, Jun Zhang, Jun Jiang

**Affiliations:** ^1^ Department of Urology, Institute of Surgery Research, Daping Hospital, Third Military Medical University, Chongqing, 400042, PR China; ^2^ Department of Bio-Medical Sciences, Philadelphia College of Osteopathic Medicine, Philadelphia, PA, 19131, USA; ^3^ Department of Pathology, Institute of Surgery Research, Daping Hospital, Third Military Medical University, Chongqing, 400042, PR China

**Keywords:** metformin, CSC, COX2, PGE2, STAT3 signaling

## Abstract

Cancer stem cells (CSCs) are a sub-population of tumor cells playing essential roles in initiation, differentiation, recurrence, metastasis and development of drug resistance of various cancers, including bladder cancer. Although multiple lines of evidence suggest that metformin is capable of repressing CSC repopulation in different cancers, the effect of metformin on bladder cancer CSCs remains largely unknown. Using the N-methyl-N-nitrosourea (MNU)-induced rat orthotropic bladder cancer model, we demonstrated that metformin is capable of repressing bladder cancer progression from both mild to moderate/severe dysplasia lesions and from carcinoma in situ (CIS) to invasive lesions. Metformin also can arrest bladder cancer cells in G1/S phases, which subsequently leads to apoptosis. And also metformin represses bladder cancer CSC repopulation evidenced by reducing cytokeratin 14 (CK14+) and octamer-binding transcription factor 3/4 (OCT3/4+) cells in both animal and cellular models. More importantly, we found that metformin exerts these anticancer effects by inhibiting COX2, subsequently PGE2 as well as the activation of STAT3. In conclusion, we are the first to systemically demonstrate in both animal and cell models that metformin inhibits bladder cancer progression by inhibiting stem cell repopulation through the COX2/PGE2/STAT3 axis.

## INTRODUCTION

Bladder cancer is the sixth most common cancer in the US [1] and about 90% of them are urothelial carcinomas with muscle-invasive lesions, papillary lesions and CIS; while the other 10% are distributed between squamous cell carcinoma (SCC) and adenocarcinoma [2, 3]. Although both CIS and papillary lesions belong to superficial urothelial carcinomas, they show different morphologies as well as invasive potentials. Therefore, it has been suggested that CIS and papillary lesions arise from different cell types or harbor distinct mutations [4–16]. Indeed, supportive evidence has shown that both CIS and invasive urothelial carcinoma arise from basal cells, whereas intermediate cells serve as the progenitors of papillary carcinomas [17]. In addition and similar to that epithelial tumors constitute heterogeneous cell populations [18], while bladder cancer comprises cells at different differentiation stages [8, 12]. Cells expressing CK14 were considered the least differentiated population, which are also known as CSC or tumor initial cells (TIC). More importantly, it has been demonstrated that elevated levels of CK14+ cells correlate with poor survival [19]. Accumulating evidence also supports the notion that CSCs are responsible for tumor initiation, relapse, metastasis, as well as chemo- and radio-resistance [20]. A recent study [21] demonstrated that ablation of basal stem cells is capable of preventing bladder cancer development. Given the fact that CSCs are mainly derived from basal stem cells, CSCs are likely to play a pivotal role in bladder cancer initiation and progression.

Although metformin is mainly considered as a antidiabetic agent [22], it has also showed anticancer effect in many cancer types, including melanoma [23], colon cancer [24], ovarian cancer [25], prostate cancer [26] and bladder cancer [27] by enhancing cancer cell apoptosis, inhibiting epithelial to mesenchymal transition (EMT) and targeting cancer stem cells. These effects were evidenced by the fact that metformin can also synergize with certain chemotherapeutic drugs [28–33]. For example, metformin not only inhibits breast tumor cell growth [34, 35] but also selectively represses the CSCs [31]. Results from bladder cancer research suggest that metformin may be capable of inhibiting cancer cell growth [36] and blocking tumor progression from precancerous to invasive tumors [37].

Upregulated cyclooxygenase-2 (COX-2), a key enzyme in prostaglandin E2 (PGE2) biosynthesis pathway, has been reported in different cancers including bladder cancer [38, 39]. In N-butyl-4-N-(4-hydroxybutyl) nitrosamine (BBN)-induced rat urinary bladder cancer model, COX-2 is also upregulated in both pre-neoplastic and neoplastic lesions [40] and elevated expression of COX-2 is correlated with higher levels of PGE2 in many cancers [41]. Furthermore, it appears that PGE2 plays important roles in CSC repopulation of different cancers including bladder cancer [42, 43]. Mechanistically, COX2/PGE2 is likely to play an important role in CSC repopulation through activating the JAK2 (Janus kinase 2)/STAT3 (signal transducer and activator of transcription 3) signaling pathway [44]. Accumulating evidence indicates that alterations in JAK/STAT pathway are involved in oncogenesis [45]. In liver tumor initiation, the STAT3-upregulated NANOG CD24+ (cluster of differentiation 24+) CSCs is believed to play an important role [46]. Finally, in Stat3-transgenic mice with BBN-induced bladder cancer, an early expansion of CK14+ stem cells has been observed [47]. In this research, we demonstrated that metformin is capable of repressing bladder cancer CSC repopulation through inhibiting the COX2/PGE2/STAT3 axis in both animal and cellular models.

## RESULTS

### Effects of metformin on bladder cancer development

**Figure 1:**
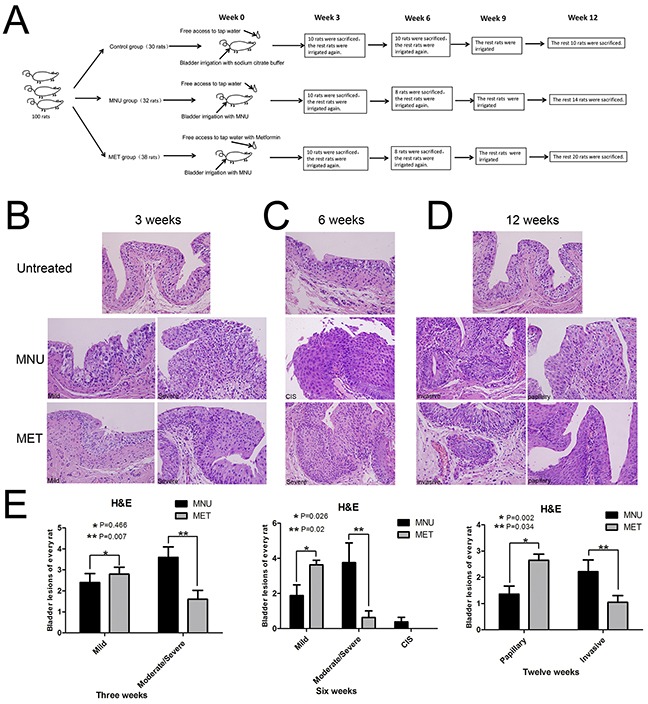
Effects of metformin on bladder cancer development **A.** The experimental scheme of cancer induction and metformin treatment. **B–D.** The effects of metformin on bladder cancer initiation and progression. Bladder tissues from the animals treated with or without metformin were collected and stained with H&E at week 3 (B), week 6 (C) and week 12 (D). **E.** The numbers of different lesions in each animal at different time points were counted and the averages of lesions in the animals treated with and without metformin were plotted. (B–D) Magnifications: X400.

We decided to use the MNU (N-methyl-N-nitrosourea)-induced rat orthotopic bladder cancer model to examine if metformin (MET) has any effect on bladder cancer initiation and progression. The experimental scheme is shown in Figure 1A and the general features of the MNU-induced bladder cancer treated with or without MET were shown in Supplementary Table S1, S2, and S3. Figure 1B–1D showed the pathological changes of bladder tumors in rat model treated with or without MET. The average numbers of lesions in animals treated with and without metformin were plotted (Figure 1E). As shown in Figure 1E (left pane) that at week 3, the total number of mild dysplasia lesions in MNU and MET groups were comparable (24 lesions/10 rats in the MNU group *vs* 28 lesions/10 rats in the MET group, *P*=0.466). However, the number of moderate/severe dysplasia lesions in animals treated with metformin (MET group, 16/10 rats) is significantly lower than that in MNU group (36/10 rats) (*P*=0.007). At week 6 (Figure 1E, middle panel), metformin treatment increased the number of mild dysplasia lesions significantly with 29 mild dysplasia lesions/8 rats in MET group compared with 15 lesions/8 rats in MNU group (*P*=0.026); but decreased the number of moderate/severe dysplasia lesions dramatically with 5 moderate/severe dysplasia in MET group compared with 30 lesions/8 rats in MNU group (*P*=0.02). In addition, 3 CIS lesions were also seen in the MNU group with none of such lesion in the MET group. At week 12 (Figure 1E, right pane), there were more papillary tumor lesions in MET group than that of MNU group (53 lesions/20 rats in MET group *vs* 19 lesions/14 rats in MNU group, *P*=0.03). However, the invasive lesions in MET group (46 invasive lesions/20 rats) are significantly less than that of MNU group (49 lesions/14 rats) (*P*=0.034). These data altogether suggest that metformin may not be able to inhibit bladder cancer initiation but be capable of repressing the progression from mild to moderate/severe dysplasia lesions and from CIS to invasive lesions.

### Metformin inhibits cancer cell growth and enhances apoptosis

**Figure 2:**
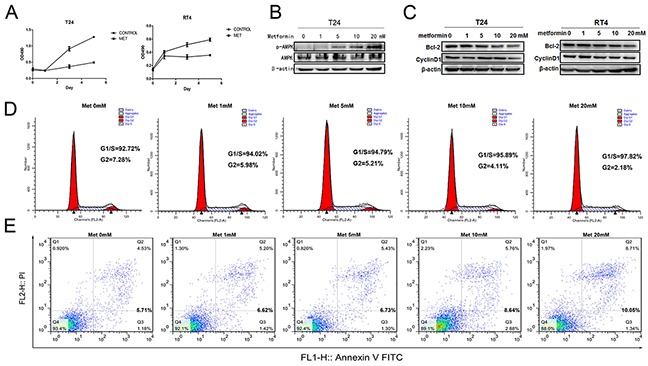
Effects of metformin on bladder cancer proliferation and apoptosis **A.** T24 and RT4 cells were seeded in 96-well plates with 0.5×10^5^ cells per well in growth media with or without metformin (20mM) and cultured for 5 days. Cell viabilities were estimated by MTT every other day. **B.** T24 cells were treated with different concentrations of metformin (0, 1, 5, 10 and 20 mM) for 24 h. The levels of total AMPK and phosphorylated AMPK were estimated by western blot assays. **C.** The effects of metformin on the levels of Bcl-2 and CyclinD1 were measured by western blotting when T24 and RT4 cells were treated with increasing concentrations of metformin. **D.** and **E.** T24 cells were treated with different concentrations of metformin and the numbers of cells at different stages of cycle were analyzed by flow cytometry (D), or stained with PI and FITC-labelled Annexin V and subsequently underwent flow cytometry analysis to determine the percentage of apoptotic cells (E).

**Figure 3:**
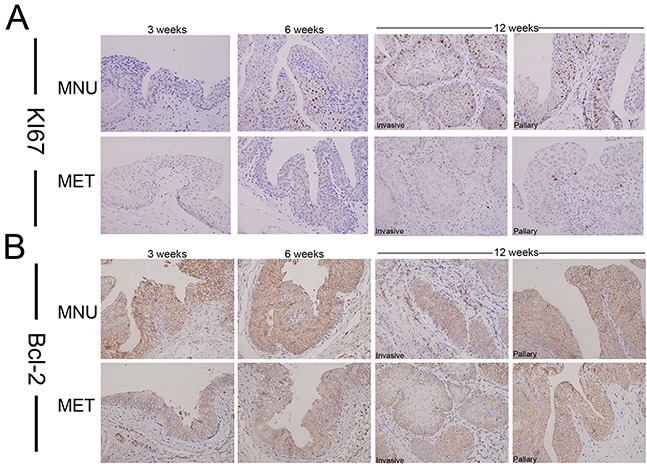
The effect of metformin on the expression of Ki67 and Bcl-2 in bladder cancers development Cancer tissues were collected from the animals treated with or without metformin and immunostaining was conducted with antibodies against Ki67 **A.** or Bcl-2 **B.** Magnifications: X400.

Next, we decided to use bladder cancer cell lines to dissect the mechanisms of metformin effects on cancer progression. To test if metformin can affect bladder cancer cell growth, we treated two bladder cancer cell lines T24 and RT4 with or without 20 mM of metformin for 5 days and the numbers of viable cells were estimated by MTT (3-(4,5-dimethylthiazol-2-yl)-2,5-diphenyl tetrazolium bromide). Figure 2A shows that metformin is able to inhibit both T24 (left panel) and RT4 (right panel) cell growth. AMPK (Adenosine Monophosphate Activated Protein Kinase) is a well-established molecular target of metformin [33, 35]. We estimated the levels of the phosphorylated AMPK in T24 cells treated with different concentrations of metformin. Figure 2B shows that metformin is capable of upregulating the phosphorylation of AMPK in a dose-dependent manner without affecting the level of total AMPK. In addition, results from western blotting assays showed that metformin is capable of repressing the levels of cyclin D1 and Bcl-2 in both cell lines in a dose-dependent manner (Figure 2C). Then, we estimated the effect of metformin on cell cycles distribution by treating T24 cells with different concentrations of metformin. Figure 2D shows that metformin treatment increased the cell populations in G1/S phases and decreased the population in G2 phase. Furthermore, the apoptotic cell populations were upregulated by metformin in a dose-dependent manner (Figure 2E). More importantly, immunohistochemical staining showed the expression of Ki67 (Figure 3A) and Bcl-2 (Figure 3B) in bladder specimens were significantly repressed by metformin treatment. Of note, not only the intensity of Ki67 but also the numbers of Ki67-positive cells were significantly reduced in metformin-treated group at all time points (Supplementary Table S4 and Figure S2). These data suggest that metformin exerts its anticancer effects by slowing down cell growth and enhancing apoptosis.

### Metformin selectively inhibits repopulation of bladder cancer stem cells

**Figure 4:**
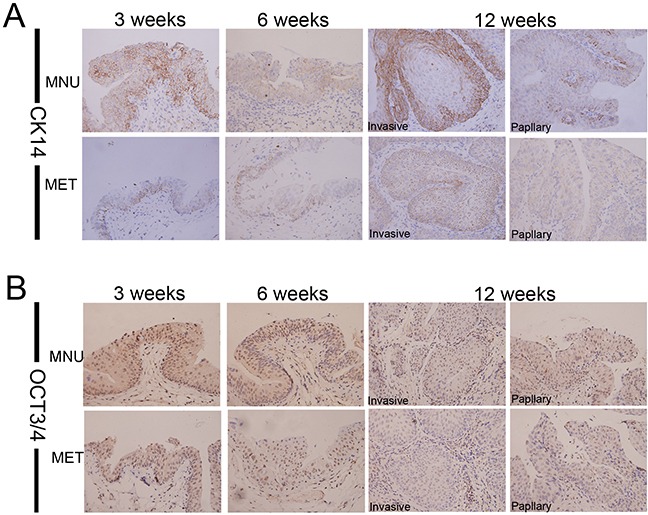
Metformin inhibits the repopulation of CSC Bladder lesions from the model animals treated with or without metformin for different periods (3, 6 and 12 weeks) were analyzed by Immunohistochemical staining with antibodies against different stem cell markers CK14 **A.** and OCT3/4 **B.** Magnifications: X400.

It has been suggested that bladder cancers arise from distinct urothelial sub-populations with CIS and invasive urothelial carcinoma arising from basal cells and papillary carcinomas deriving from intermediate cells. In order to determine if metformin exerts distinct effect on different precursor cells, we examined the expression of two stem cell markers, CK14 and OCT3/4 in tumors derived from animals treated with or without metformin. As shown in Figure 4 that metformin treatment reduced the number of CK14- (Figure 4A) and OCT3/4-positive cells (Figure 4B, Supplementary Table S4 and Supplementary Figure S2). On the other hand, at all time points examined (3, 6 and 12 weeks) more CK20-positive cells were seen in the tissues of the animals treated with metformin (Supplementary Figure S1). It appears that metformin selectively inhibits CIS and invasive lesions without affecting papillary lesions. These data suggest that metformin may inhibit the repopulation of basal stem cell and subsequently repress early CIS and invasive cancer progression.

### Metformin inhibits COX2/PGE2/STAT3 axis by downregulating COX2

**Figure 5:**
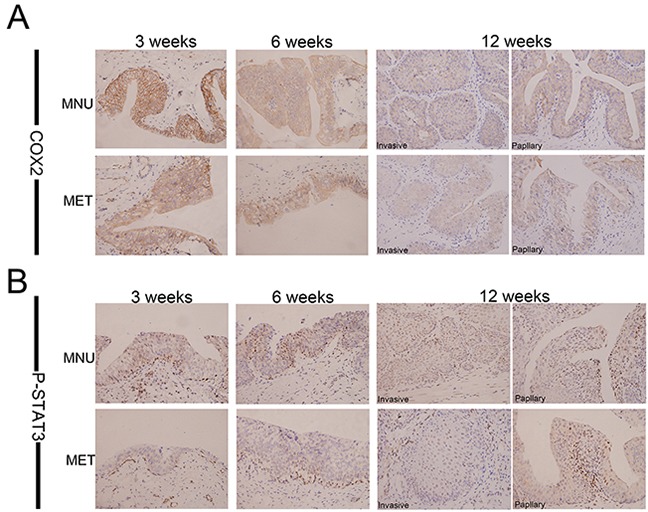
Metformin inhibits expression of COX2 and P-STAT3 *in vivo* Immunohistochemical analyses of bladder lesions including mild, severe hyperplasia, invasive and papillary lesions from the rats of both MNU and MET groups in the sections with COX2 **A.** and P-STAT3 **B.** at 3, 6,12 weeks. Magnifications: X400.

**Figure 6:**
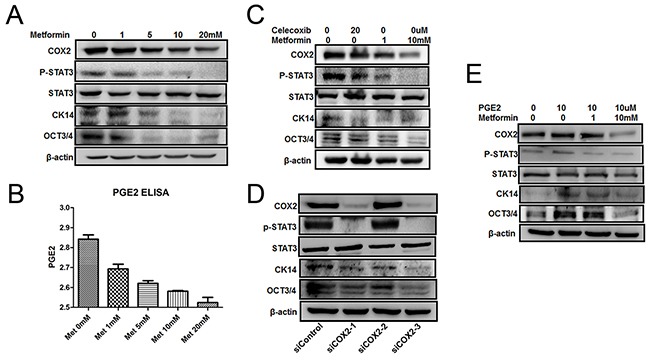
Effect of metformin on COX2/PGE2/STAT3 axis **A.** and **B.** T24 cell was seeded in 6-well plates and serum-starved for 24 h before exposed to metformin (0, 1, 5, 10, and 20 mM) for 24 h. Cell lysates were assayed by western blot with antibodies against CK14, OCT3/4, COX2, P-STAT3 or STAT3 (A), and the culture media were collect for ELISA assays (B). **C, D.** and **E.** T24 cells were treated with either celecoxib (20 μM) or metformin (1, 10 mM) for 24 h (C), and transfected with three siRNAs against COX2 and one for negative control and the cells were cultured for 48 h (D), The cells were treated by PGE2 (10 μM) with or without metformin (1, 10 mM) for 24 h (E). Cell lysates were assayed by western blot with antibodies against CK14, OCT3/4, COX2, P-STAT3 or STAT3.

Multiple lines of evidence suggest that PGE2 is an important inducer of CSC repopulation in different cancers including bladder cancer [42]. To dissect the mechanism of metformin inhibition of CK14- and OCT3/4-positive cell repopulation, we examined the effect of metformin on COX2, a key enzyme catalyzing the biosynthesis of PGE2. Figure 5A shows that metformin is capable of repressing the expression of COX2 at all time points in both papillary and invasive lesions. These observations were further substantiated by the dose-dependent inhibition of COX2 *in vitro* when T24 cells were treated with different concentrations of metformin (Figure 6A). Consistent with COX2 inhibition, the levels of PGE2 in culture media were significantly lower when the cells were treated with metformin (Figure 6B). Based on the facts that in colorectal cancer the JAK2/STAT3 signaling pathway is regulated by PGE2 [44], and STAT3 is not only involved in the expansion of CK14-positive stem cells in bladder cancer [47], but STAT3 activation in urothelial stem cells also leads to invasive bladder cancer progression, we decided to examine if metformin-inhibited bladder cancer development is through the COX2/PGE2/STAT3 axis. Immunohistochemistry (Figure 5B, Supplementary Table S4 and Supplementary Figure S2) shows the levels of p-STAT3 in the bladder cancer animal model treated with metformin are consistently lower. These observations were further substantiated by the dose-dependent downregulation of phosphorylated STAT3 in cellular model (Figure 6A). Of note, metformin treatment has no effect on the levels of total STAT3. Consistent with downregulation of the levels of COX2 and PGE2 as well as inhibition of STAT3 phosphorylation/activation, the levels of both CK14 and OCT3/4 were repressed by metformin in a dose-dependent manner (Figure 6A); suggesting metformin may repress bladder cancer development through the COX2/STAT3 pathway.

To further substantiate our hypothesis, we treated T24 cells with either a COX2 specific inhibitor celecoxib, or different concentrations of metformin. As showed in Figure 6C, metformin repressed all above-mentioned factors as celecoxib did. In addition, we knocked down COX2 in T24 cells by siRNA technique and monitored the changes of the molecules including COX2, STAT3, p-STAT3, CK14 and OCT3/4. As shown in Figure 6D, both siCOX2-1 and siCOX2-3 knocked down COX2 efficaciously but for some unknown reason that siCOX2-2 failed to knock down COX2. When COX2 is knocked down (lanes 2 and 4 in Figure 6D), all three markers p-STAT3, CK14 and OCT3/4 were downregulated significantly. However, the total STAT3 levels were not affected at all. To demonstrate that metformin affects CSC repopulation through PGE2, we treated the cells with metformin in the presence or absence of exogenously added PGE2. Figure 6E shows that exogenously added PGE2 is able to counteract metformin and upregulate all CSC markers tested without affecting the levels of COX2; demonstrating the essentiality of PGE2 in bladder cancer stem cell repopulation. These data collectively suggest that metformin represses CK14+ stem cell repopulation by downregulating COX2 and subsequently inhibiting the COX2/PGE2/STAT3 axis. However, we noticed that high concentration (10mM) of metformin is capable of inhibiting the levels of both CK14 and OCT3/4 by directly repressing STAT3 phosphorylation/activation even in the presence of exogenous PGE2, a phenomenon has been observed previously [37]. This suggests that metformin may also be able to repress bladder cancer development through a COX2-independent manner.

## DISCUSSION

**Figure 7:**
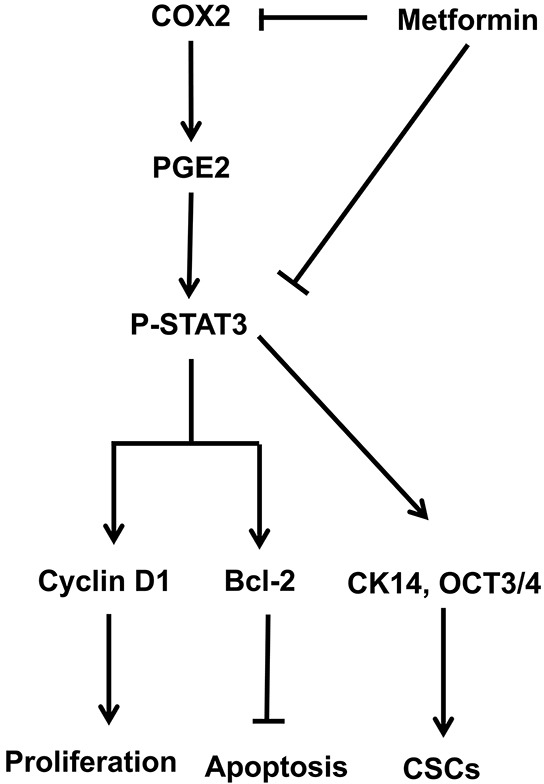
Schematic model of the hypothesized mechanism by which metformin inhibits bladder cancer

Using the MNU-induced rat orthotopic bladder cancer model we demonstrated the anti-tumor effects of metformin and explored its underlying mechanism with experiments in bladder cancer cell lines. Data from both *in vitro* and *in vivo* experiments suggest that metformin may not be able to prevent bladder cancer initiation but it appeared to be capable of slowing down the progression both from mild to moderate/severe dysplasia lesions and from CIS lesions to invasive lesions. By directly downregulating COX2, metformin is able to downregulate the level of PGE2, which subsequently leads to reduced STAT3 phosphorylation/activation. Attenuated STAT3 signaling pathway results in reduced levels of cyclinD1 and Bcl-2, which caused cancer cells being arrested in G1/S phases and ultimately apoptotic cell death. Based on these findings, we proposed that metformin suppressed bladder cancer development by inhibiting cancer stem cell repopulation via the COX2/PGE2/STAT3 axis (Figure 7).

Carcinogen-induced rat bladder tumor is a well-established model system, which has been widely used in bladder cancer researches for decades. BBN (N-butyl-N-(4-hydroxybutyl) nitrosamine), MNU (N-methyl-N-nitrosourea) and FANFT (N-[4-(5-nitro-2-furyl)-2-thiazolyl]-formamide) are the most commonly-used carcinogenic inducers. Compared to the models induced by BBN and FANFT, both of them must be taken orally, establishing the MNU-induced model is much easier, quicker and cheaper [48]. On the other hand, comparing to cell transplantation model, tumors in animal models more closely represent human bladder tumors in histology, biochemical properties, molecular and genetic characteristics, natural history and biological behavior. In addition, the carcinogen-induced tumor is a more ideal system for studying both tumor initiation and progression including dysplasia, CIS, papillary and invasive cancers. It is the animal model that enabled us to differentiate the effects of metformin on tumor progression from initiation. Therefore, we believe that this model system would also be more ideal in studying the pathogenesis, prevention and potential bladder cancer therapies.

It has been noticed recently that the anticancer effect of metformin may rely on its role in CSC inhibition [49]. Metformin inhibits CSCs repopulation in different cancers including colon cancer [50], pancreatic [51], esophageal cancer [52], skin cancer [53] and ovarian cancer [54]. CSCs are a sub-population of cancer cells responsible for tumor initiation, differentiation, recurrence, metastasis, and drug resistance [20]. Patients with muscle-invasive bladder cancer showed higher recurrence rate and poor survival. Furthermore, it is believed that CSCs play important roles in the development of resistance to multiple chemotherapies [20]. There are many well-established CSC markers, including cytokeratin 5 (CK5), cytokeratin 14 (CK14) and aldehyde dehydrogenase 1 family, member A1 (ALDH1A1). In human bladder cancer, the primitive stem cell marker CK14 also serves as the precursor of CK5 [12]. This means that CK14+ cells are scattered and represent only a subpopulation of CK5+ urothelial cells. We found that metformin is able to dose-dependently repress CK14+ cells in animal model and downregulate the levels of CK14 in bladder cancer cell lines. To our knowledge, we are the first to systemically demonstrate that metformin inhibits bladder cancer development by directly targeting CSC repopulation.

It has been well-established that metformin plays important roles in different signaling pathways, including AMPK/mTOR [36], TGF-β/IL-6 [56], Wnt/β-catenin [57]; autophagy pathway [58], EMT [30]. The current body of evidence is inadequate to establish an absolute protective role of metformin in bladder cancer [59]. But in consistence with that reported by Pan et al [37], our results support the notion that metformin represses bladder cancer development through STAT3 signaling pathway. In addition to that, we demonstrated that metformin inhibits bladder cancer cell growth and enhances apoptosis by downregulating cyclinD1 and Bcl-2. Also consistent with that reported by Zhang et al [36], we showed that metformin dose-dependently arrests bladder cancer cells in G1/S phases and subsequent apoptosis. Based on the fact that (1) metformin is able to inhibit COX2 [60]; (2) PGE2, a product catalyzed by COX2, is capable of promoting stem cell expansion [61] and mobilization [62]; and (3) blocking PGE2 abrogates bladder cancer chemo-resistance by repressing CK14+ cells repopulation [62], we explored the possibility of metformin effect on COX2/PGE2 pathway and found that metformin is indeed capable of downregulating COX2 and subsequently reducing the level of PGE2. Since STAT3, a downstream target of COX2/PGE2, is involved in the regulation of primitive CK14+ cell expansion [47], we propose that in bladder cancer metformin inhibits CK14+ cell repopulation through the COX2/PGE2/STAT3 axis.

In conclusion, to our best knowledge that we are the first to use both cell and animal models to systemically demonstrate that metformin inhibits bladder cancer progression by inhibiting cancer stem cell repopulation through the COX2/PGE2/STAT3 axis. Given the fact that metformin is capable of repressing bladder cancer progression, administration of metformin to bladder cancer patients may extend therapeutic period for application of other cancer treatments.

## MATERIALS AND METHODS

### Metformin and N-methyl-N-nitrosourea (MNU) treatment protocol

Adult female SD rats at 5-6 weeks of age were obtained from Animal Experimental Center of Daping Hospital, Third Military Medical University (Chongqing, China) and housed in the research center. Animal experiments in this study were approved by the Institutional Animal Care and Use Committee of Third Military Medical University. Animals were fed with standard laboratory rat chow with free access of tap water. Rats were divided into three groups: control group; N-methyl-N-nitrosourea-treated group (MNU group); and MNU + metformin (MET group) (Figure 1A). For MNU treatment, 2.5 mg of MNU (SICHUAN HANOVI TECHNOLOGY) dissolved in sodium citrate buffer (5 g/L) was irrigated into the rat bladders via epidural catheter every 3 weeks (i.e. week 0, 3, 6, 9). The rats in MET group access tap water with metformin (1 g/L) freely. Rats were sacrificed at week 3 (n=30), week 6 (n=26), and week 12 (n=44), respectively. Blood was drawn from each rat and used for glucose measurement. The bladders were fixed in 10% formalin and paraffin embedded for hematoxylin and eosin (H&E) and immunostaining examination was conducted by Hualiang Xiao, a certificated pathologist.

### Immunostaining

Tumor specimens were fixed in 10% formaldehyde solution and embedded in paraffin, and sections were mounted onto glass slides. Sections were then deparaffinized in xylene, re-hydrated through ethanol, and heated for 30 min to enhance the heat-induced antigen retrieval. To block non-specific reactions, slides were blocked in respective serum at 4°C overnight. Primary antibodies against Ki67 (1:300 Origene), CK14(1:100 Abcam) and CK20 (1:400 Abcam), OCT3/4 (1:200 Santa Cruz Biotech-nology), COX2 (1:400 Origene), p-STAT3(Tyr705) (1:100 Cell signaling) and Bcl-2 (1:100 Santa Cruz Biotech-nology) were used. Tissue sections were incubated with each antibody overnight at 4°C, and then incubated with horseradish peroxidase-conjugated anti-rabbit, anti-mouse or anti-goat IgG secondary antibodies. Slides were subsequently treated with a streptavidinperoxidase reagent and incubated in phosphate-buffered salinediaminobenzidine and 1% hydrogen peroxide, followed by counterstaining with Mayer's haematoxylin.

### Cell culture and viability assay (MTT)

Human bladder cancer cells (T24 and RT4) were obtained from Cell Bank of Shanghai Institiutes for biological Sciences (Chinese Academy of Sciences), and cultured in DMEM, High Glucose (Hyclone) supplemented with 10% FBS (Gibico) at 37°C with 5% carbon dioxide. Viability assays were conducted with 3-(4,5-dimethylthiazol-2-yl)-2,5-diphenyl tetrazolium bromide (MTT) dye (5 mg/ml, Sigma). The density of the solubilized formazan was read at 490 nm spectrophotometrically (Bio-Rad, Hercules, and CA)

### Analysis of cell cycle and apoptosis

T24 Cells were seeded in 75 cm^2^ flasks and serum-starved for 24 h. Metformin was added to the culture to final concentrations of 0, 1, 5, 10, 20 mM and incubated for 24 h. Cells were washed with cold PBS for three times, resuspended in 70% ethanol used for analysis of cell cycle distribution, or directly Annexin V binding buffer used for analysis of apoptosis status.

### Western blotting

T24 or RT4 cells were seeded in 6-well plates 2×10^5^ cells/well, treated with corresponding reagents, including metformin (0, 1 mM, 5 mM, 10 mM, 20 mM), Celecoxib (20 μM YUANYE BioTECH, Shanghai, China) and PGE2(10 μM, Sigma). Cell lysates were separated on SDS-PAGE followed by western blotting assay as described [26] with the following primary antibodies: Bcl-2 (1:1000 Epitomics), Cyclin D1 (1:1000 Fantibody, China), CK14 (1:400 Abcam), STAT3 (1:2000 Abcam), OCT3/4 (1:400 Santa Cruz Biotech-nology), COX2 (1:2000 Origene), p-STAT3 (Tyr705) (1:2000 Cell Signaling), AMPK (1:700 Proteintech), p-AMPK (1:1000 Cell Signaling) and β-actin (1:5000 Cell Signaling).

### PGE2 ELISA assay

T24 Cells were seeded in 6-well plates and serum-starved for 24 h. Different concentrations (0, 1, 5, 10, 20 mM) of metformin was added into the media and cultured for 24 hours. The supernatant was collected and prostaglandin E2 (PGE2) released to the culture media was measured using commercially available enzyme-linked immunosorbent assay (ELISA) kits from Cloud-clone corp.

### COX-2 siRNA transfection

As reported previously [63], the following primers were obtained from Shanghai Genechem Co., LTD (China): scramble siRNA (MOCK) sense sequence 5′-CCU ACG CCA CCA AUU UCG U-3′, antisense sequence 5′-ACG AAA UUG GUG GCG UAG G-3′; and COX-2 siRNA: siCOX2-1 sense sequence 5′-CAG AUG AAA UAC CAG UCU U-3′, antisense sequence 5′-AAG ACUGGU AUU UCA UCU G-3′; siCOX2-2 sense sequence 5′-GAG GUU AAU GAA GUA CCA A-3′,, antisense sequence 5′-UUG GUA CUU CAU UAA CCU C-3′; and siCOX2-3 sense sequence 5′-CAC CAA GAG UAU AAA CCU U-3′, antisense sequence 5′-AAG GUU UAU ACU CUU GGU G-3′. T24 cells were plated at 80% confluence in 6-well plates and then starved with serum deprivation for 24 h. Before transfection, diluted 5 μl lipo2000 was added into diluted siRNA, and then the mix was added into the medium. The cells were transfected for 48 h, then lysed for western blot.

### Statistical analysis

GraphPad Prism 5.0 was used for all statistical analyses. Data were presented as means±Standard deviation (SD). In all of the tests, a *P*-value of less than 0.05 was considered statistically significant.

## SUPPLEMENTARY FIGURES AND TABLES



## References

[1] Siegel R, Naishadham D, Jemal A (2013). Cancer statistics, 2013. CA Cancer J Clin.

[2] Prasad SM, Decastro GJ, Steinberg GD (2011). Urothelial carcinoma of the bladder: Definition, treatment and future efforts. Nat Rev Urol.

[3] Dahm P, Gschwend JE (2003). Malignant non-urothelial neoplasms of the urinary bladder: A review. Eur Urol.

[4] Goebell PJ, Knowles MA (2010). Bladder cancer or bladder cancers? Genetically distinct malignant conditions of the urothelium. Urol Oncol.

[5] Wu XR (2005). Urothelial tumorigenesis: A tale of divergent pathways. Nat Rev Cancer.

[6] Knowles MA (2008). Bladder cancer subtypes defined by genomic alterations. Scand J Urol Nephrol Suppl.

[7] Castillo-Martin M, Domingo-Domenech J, Karni-Schmidt O, Matos T, Cordon-Cardo C (2010). Molecular pathways of urothelial development and bladder tumorigenesis. Urol Oncol.

[8] Chan KS, Espinosa I, Chao M, Wong D, Ailles L, Diehn M, Gill H, Presti JJ, Chang HY, van de Rijn M, Shortliffe L, Weissman IL (2009). Identification, molecular characterization, clinical prognosis, and therapeutic targeting of human bladder tumor-initiating cells. Proc Natl Acad Sci U S A.

[9] Cordon-Cardo C (2008). Molecular alterations associated with bladder cancer initiation and progression. Scand J Urol Nephrol Suppl.

[10] Chan KS, Volkmer JP, Weissman I (2010). Cancer stem cells in bladder cancer: A revisited and evolving concept. Curr Opin Urol.

[11] Dancik GM, Owens CR, Iczkowski KA, Theodorescu D (2014). A cell of origin gene signature indicates human bladder cancer has distinct cellular progenitors. Stem Cells.

[12] Volkmer JP, Sahoo D, Chin RK, Ho PL, Tang C, Kurtova AV, Willingham SB, Pazhanisamy SK, Contreras-Trujillo H, Storm TA, Lotan Y, Beck AH, Chung BI (2012). Three differentiation states risk-stratify bladder cancer into distinct subtypes. Proc Natl Acad Sci U S A.

[13] Dalbagni G, Presti J, Reuter V, Fair WR, Cordon-Cardo C (1993). Genetic alterations in bladder cancer. Lancet.

[14] Spruck CR, Ohneseit PF, Gonzalez-Zulueta M, Esrig D, Miyao N, Tsai YC, Lerner SP, Schmutte C, Yang AS, Cote R, Et A (1994). Two molecular pathways to transitional cell carcinoma of the bladder. Cancer Res.

[15] Cheng L, Cheville JC, Neumann RM, Bostwick DG (2000). Flat intraepithelial lesions of the urinary bladder. Cancer.

[16] Cheng L, Darson M, Cheville JC, Neumann RM, Zincke H, Nehra A, Bostwick DG (1999). Urothelial papilloma of the bladder. Clinical and biologic implications. Cancer.

[17] Van Batavia J, Yamany T, Molotkov A, Dan H, Mansukhani M, Batourina E, Schneider K, Oyon D, Dunlop M, Wu XR, Cordon-Cardo C, Mendelsohn C (2014). Bladder cancers arise from distinct urothelial sub-populations. Nat Cell Biol.

[18] Heppner GH (1984). Tumor heterogeneity. Cancer Res.

[19] Ho PL, Kurtova A, Chan KS (2012). Normal and neoplastic urothelial stem cells: Getting to the root of the problem. Nat Rev Urol.

[20] Tran MN, Goodwin JG, McConkey DJ, Kamat AM (2010). Bladder cancer stem cells. Curr Stem Cell Res Ther.

[21] Shin K, Lim A, Odegaard JI, Honeycutt JD, Kawano S, Hsieh MH, Beachy PA (2014). Cellular origin of bladder neoplasia and tissue dynamics of its progression to invasive carcinoma. Nat Cell Biol.

[22] Goodarzi MO, Bryer-Ash M (2005). Metformin revisited: Re-evaluation of its properties and role in the pharmacopoeia of modern antidiabetic agents. Diabetes Obes Metab.

[23] Tomic T, Botton T, Cerezo M, Robert G, Luciano F, Puissant A, Gounon P, Allegra M, Bertolotto C, Bereder JM, Tartare-Deckert S, Bahadoran P, Auberger P (2011). Metformin inhibits melanoma development through autophagy and apoptosis mechanisms. Cell Death Dis.

[24] Buzzai M, Jones RG, Amaravadi RK, Lum JJ, DeBerardinis RJ, Zhao F, Viollet B, Thompson CB (2007). Systemic treatment with the antidiabetic drug metformin selectively impairs p53-deficient tumor cell growth. Cancer Res.

[25] Patel S, Singh N, Kumar L (2015). Evaluation of effects of metformin in primary ovarian cancer cells. Asian Pac J Cancer Prev.

[26] Wang Y, Liu G, Tong D, Parmar H, Hasenmayer D, Yuan W, Zhang D, Jiang J (2015). Metformin represses androgen-dependent and androgen-independent prostate cancers by targeting androgen receptor. Prostate.

[27] Nayan M, Bhindi B, Yu JL, Hermanns T, Mohammed A, Hamilton RJ, Finelli A, Jewett MA, Zlotta AR, Fleshner NE, Kulkarni GS (2015). The effect of metformin on cancer-specific survival outcomes in diabetic patients undergoing radical cystectomy for urothelial carcinoma of the bladder. Urol Oncol.

[28] Liu B, Fan Z, Edgerton SM, Deng XS, Alimova IN, Lind SE, Thor AD (2009). Metformin induces unique biological and molecular responses in triple negative breast cancer cells. Cell Cycle.

[29] Zhuang Y, Miskimins WK (2011). Metformin induces both caspase-dependent and poly(ADP-ribose) polymerase-dependent cell death in breast cancer cells. Mol Cancer Res.

[30] Cufi S, Vazquez-Martin A, Oliveras-Ferraros C, Martin-Castillo B, Joven J, Menendez JA (2010). Metformin against TGFbeta-induced epithelial-to-mesenchymal transition (EMT): From cancer stem cells to aging-associated fibrosis. Cell Cycle.

[31] Hirsch HA, Iliopoulos D, Tsichlis PN, Struhl K (2009). Metformin selectively targets cancer stem cells, and acts together with chemotherapy to block tumor growth and prolong remission. Cancer Res.

[32] Iliopoulos D, Hirsch HA, Struhl K (2011). Metformin decreases the dose of chemotherapy for prolonging tumor remission in mouse xenografts involving multiple cancer cell types. Cancer Res.

[33] Rocha GZ, Dias MM, Ropelle ER, Osorio-Costa F, Rossato FA, Vercesi AE, Saad MJ, Carvalheira JB (2011). Metformin amplifies chemotherapy-induced AMPK activation and antitumoral growth. Clin Cancer Res.

[34] Alimova IN, Liu B, Fan Z, Edgerton SM, Dillon T, Lind SE, Thor AD (2009). Metformin inhibits breast cancer cell growth, colony formation and induces cell cycle arrest in vitro. Cell Cycle.

[35] Zakikhani M, Dowling R, Fantus IG, Sonenberg N, Pollak M (2006). Metformin is an AMP kinase-dependent growth inhibitor for breast cancer cells. Cancer Res.

[36] Zhang T, Guo P, Zhang Y, Xiong H, Yu X, Xu S, Wang X, He D, Jin X (2013). The antidiabetic drug metformin inhibits the proliferation of bladder cancer cells in vitro and in vivo. Int J Mol Sci.

[37] Pan Q, Yang GL, Yang JH, Lin SL, Liu N, Liu SS, Liu MY, Zhang LH, Huang YR, Shen RL, Liu Q, Gao JX, Bo JJ (2015). Metformin can block precancerous progression to invasive tumors of bladder through inhibiting STAT3-mediated signaling pathways. J Exp Clin Cancer Res.

[38] Ristimaki A, Nieminen O, Saukkonen K, Hotakainen K, Nordling S, Haglund C (2001). Expression of cyclooxygenase-2 in human transitional cell carcinoma of the urinary bladder. Am J Pathol.

[39] Shirahama T, Sakakura C (2001). Overexpression of cyclooxygenase-2 in squamous cell carcinoma of the urinary bladder. Clin Cancer Res.

[40] Kitayama W, Denda A, Okajima E, Tsujiuchi T, Konishi Y (1999). Increased expression of cyclooxygenase-2 protein in rat urinary bladder tumors induced by N-butyl-N-(4-hydroxybutyl) nitrosamine. Carcinogenesis.

[41] Hammam OA, Aziz AA, Roshdy MS, Abdel HA (2008). Possible role of cyclooxygenase-2 in schistosomal and non-schistosomal-associated bladder cancer. Medscape J Med.

[42] Kurtova AV, Xiao J, Mo Q, Pazhanisamy S, Krasnow R, Lerner SP, Chen F, Roh TT, Lay E, Ho PL, Chan KS (2015). Blocking PGE2-induced tumour repopulation abrogates bladder cancer chemoresistance. Nature.

[43] Rudnick JA, Arendt LM, Klebba I, Hinds JW, Iyer V, Gupta PB, Naber SP, Kuperwasser C (2011). Functional heterogeneity of breast fibroblasts is defined by a prostaglandin secretory phenotype that promotes expansion of cancer-stem like cells. PLoS One.

[44] Liu X, Ji Q, Ye N, Sui H, Zhou L, Zhu H, Fan Z, Cai J, Li Q (2015). Berberine inhibits invasion and metastasis of colorectal cancer cells via COX-2/PGE2 mediated JAK2/STAT3 signaling pathway. PLoS One.

[45] Saxena NK, Sharma D, Ding X, Lin S, Marra F, Merlin D, Anania FA (2007). Concomitant activation of the JAK/STAT, PI3K/AKT, and ERK signaling is involved in leptin-mediated promotion of invasion and migration of hepatocellular carcinoma cells. Cancer Res.

[46] Lee TK, Castilho A, Cheung VC, Tang KH, Ma S, Ng IO (2011). CD24(+) liver tumor-initiating cells drive self-renewal and tumor initiation through STAT3-mediated NANOG regulation. Cell Stem Cell.

[47] Ho PL, Lay EJ, Jian W, Parra D, Chan KS (2012). Stat3 activation in urothelial stem cells leads to direct progression to invasive bladder cancer. Cancer Res.

[48] Steinberg GD, Brendler CB, Ichikawa T, Squire RA, Isaacs JT (1990). Characterization of an N-methyl-N-nitrosourea-induced autochthonous rat bladder cancer model. Cancer Res.

[49] Vazquez-Martin A, Lopez-Bonetc E, Cufi S, Oliveras-Ferraros C, Del BS, Martin-Castillo B, Menendez JA (2011). Repositioning chloroquine and metformin to eliminate cancer stem cell traits in pre-malignant lesions. Drug Resist Updat.

[50] Montales MT, Simmen RC, Ferreira ES, Neves VA, Simmen FA (2015). Metformin and soybean-derived bioactive molecules attenuate the expansion of stem cell-like epithelial subpopulation and confer apoptotic sensitivity in human colon cancer cells. Genes Nutr.

[51] Sancho P, Burgos-Ramos E, Tavera A, Bou KT, Jagust P, Schoenhals M, Barneda D, Sellers K, Campos-Olivas R, Grana O, Viera CR, Yuneva M, Sainz BJ (2015). MYC/PGC-1alpha balance determines the metabolic phenotype and plasticity of pancreatic cancer stem cells. Cell Metab.

[52] Honjo S, Ajani JA, Scott AW, Chen Q, Skinner HD, Stroehlein J, Johnson RL, Song S (2014). Metformin sensitizes chemotherapy by targeting cancer stem cells and the mTOR pathway in esophageal cancer. Int J Oncol.

[53] Reddi A, Powers MA, Dellavalle RP (2014). Therapeutic potential of the anti-diabetic agent metformin in targeting the skin cancer stem cell diaspora. Exp Dermatol.

[54] Shank JJ, Yang K, Ghannam J, Cabrera L, Johnston CJ, Reynolds RK, Buckanovich RJ (2012). Metformin targets ovarian cancer stem cells in vitro and in vivo. Gynecol Oncol.

[55] Yin B, Zeng Y, Liu G, Wang X, Wang P, Song Y (2014). MAGE-A3 is highly expressed in a cancer stem cell-like side population of bladder cancer cells. Int J Clin Exp Pathol.

[56] Li L, Han R, Xiao H, Lin C, Wang Y, Liu H, Li K, Chen H, Sun F, Yang Z, Jiang J, He Y (2014). Metformin sensitizes EGFR-TKI-resistant human lung cancer cells in vitro and in vivo through inhibition of IL-6 signaling and EMT reversal. Clin Cancer Res.

[57] Banerjee P, Dutta S, Pal R (2016). Dysregulation of Wnt-signaling and a candidate set of miRNAs underlie the effect of metformin on neural crest cell development. Stem Cells.

[58] Takahashi A, Kimura F, Yamanaka A, Takebayashi A, Kita N, Takahashi K, Murakami T (2014). Metformin impairs growth of endometrial cancer cells via cell cycle arrest and concomitant autophagy and apoptosis. Cancer Cell Int.

[59] Spradling K, Youssef RF (2015). Controversies related to diabetes and risk of bladder cancer. Mini Rev Med Chem.

[60] Luchetti CG, Miko E, Szekeres-Bartho J, Paz DA, Motta AB (2008). Dehydroepiandrosterone and metformin modulate progesterone-induced blocking factor (PIBF), cyclooxygenase 2 (COX2) and cytokines in early pregnant mice. J Steroid Biochem Mol Biol.

[61] Goessling W, North TE, Loewer S, Lord AM, Lee S, Stoick-Cooper CL, Weidinger G, Puder M, Daley GQ, Moon RT, Zon LI (2009). Genetic interaction of PGE2 and Wnt signaling regulates developmental specification of stem cells and regeneration. Cell.

[62] Hoggatt J, Mohammad KS, Singh P, Hoggatt AF, Chitteti BR, Speth JM, Hu P, Poteat BA, Stilger KN, Ferraro F, Silberstein L, Wong FK, Farag SS (2013). Differential stem- and progenitor-cell trafficking by prostaglandin E2. Nature.

[63] Park JS, Yang HN, Jeon SY, Woo DG, Kim MS, Park KH (2012). The use of anti-COX2 siRNA coated onto PLGA nanoparticles loading dexamethasone in the treatment of rheumatoid arthritis. Biomaterials.

