# Supplementary material for: Metformin represses bladder cancer progression by inhibiting stem cell repopulation via COX2/PGE2/STAT3 axis
Source: Oncotarget. 2016 Apr 5;7(19):28235–46. doi: 10.18632/oncotarget.8595 (PMC5053723; doi:10.18632/oncotarget.8595)
Supplement: Supplementary file 1 [file oncotarget-07-28235-s001.pdf]

SUPPLEMENTARY FIGURES AND TABLES

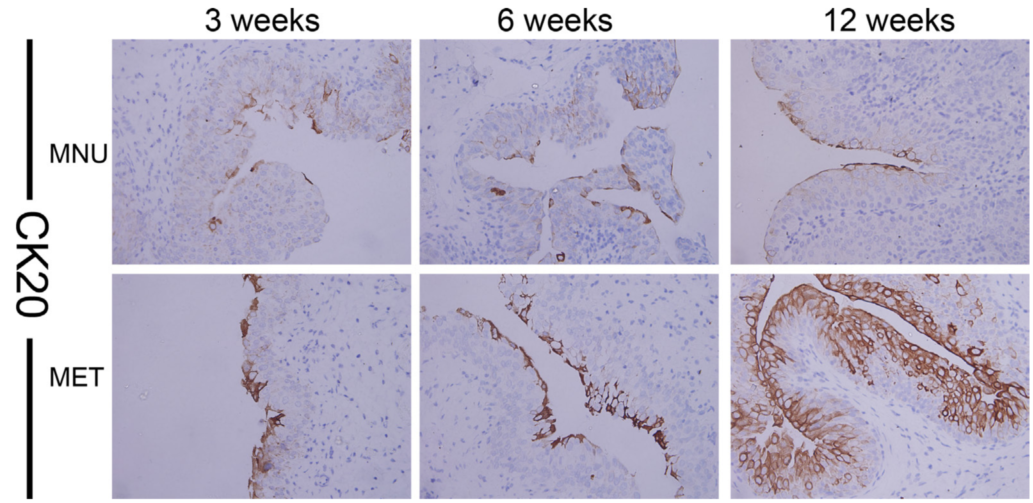

Supplementary Figure S1: Immunohistochemical analysis of CK20 in bladder lesions from the rats of both MNU and MET groups.

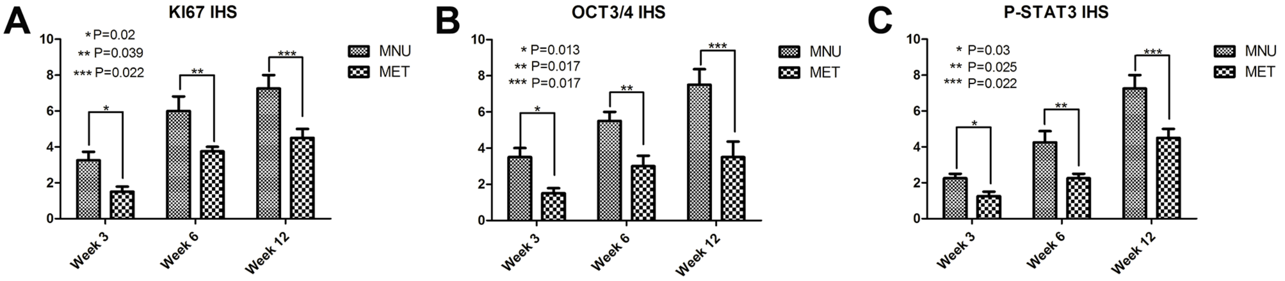

Supplementary Figuer S2: The Immunohistochemical scores(IHS) of both MNU and MET groups at week 3, 6, 12.

Supplementary Table S1: The general features of the MNU-induced bladder cancer treated with or without MET at week 3

NO.	Group	Weight (g)	Glucose (mmol/L)	Hyperplasia/Dysplasia			Papillary	CIS	T1/T2	SCC
				Mild	Moderate	Severe				
1	Control	185	7.9	0	0	0	0	0	0	0
2	Control	189	8.0	0	0	0	0	0	0	0
3	Control	185	8.1	0	0	0	0	0	0	0
4	Control	188	7.9	0	0	0	0	0	0	0
5	Control	182	7.4	0	0	0	0	0	0	0
6	Control	182	7.8	0	0	0	0	0	0	0
7	Control	183	8.1	0	0	0	0	0	0	0
8	Control	180	8.0	0	0	0	0	0	0	0
9	Control	185	7.5	0	0	0	0	0	0	0
10	Control	183	8.0	0	0	0	0	0	0	0
1	MNU	170	10.0	2	0	3	0	0	0	0
2	MNU	174	9.8	4	0	2	0	0	0	0
3	MNU	170	10.5	1	4	2	0	0	0	0
4	MNU	201	11.1	3	2	1	0	0	0	0
5	MNU	200	11.8	2	4	1	0	0	0	0
6	MNU	176	6.6	0	3	3	0	0	0	0
7	MNU	175	7.8	4	2	0	0	0	0	0
8	MNU	185	7.5	2	3	0	0	0	0	0
9	MNU	189	5.3	4	2	0	0	0	0	0
10	MNU	187	7.0	2	3	1	0	0	0	0
1	MET	205	10.6	2	1	0	0	0	0	0
2	MET	208	8.1	3	1	0	0	0	0	0
3	MET	154	9.4	3	1	3	0	0	0	0
4	MET	187	8.8	3	3	0	0	0	0	0
5	MET	170	8.0	3	1	0	0	0	0	0
6	MET	173	6.3	5	0	0	0	0	0	0
7	MET	160	8.1	3	1	0	0	0	0	0
8	MET	184	6.5	3	0	0	0	0	0	0
9	MET	204	9.7	2	2	0	0	0	0	0
10	MET	209	8.1	1	3	0	0	0	0	0

Supplementary Table S2: The general features of the MNU-induced bladder cancer treated with or without MET at week 6

NO.	Group	Weight (g)	Glucose (mmol/L)	Hyperplasia/Dysplasia			Papillary	CIS	T1/T2	SCC
				Mild	Moderate	Severe				
1	Control	210	10.0	0	0	0	0	0	0	0
2	Control	218	10.0	0	0	0	0	0	0	0
3	Control	213	10.0	0	0	0	0	0	0	0
4	Control	219	8.9	0	0	0	0	0	0	0
5	Control	220	8.9	0	0	0	0	0	0	0
6	Control	211	9.0	0	0	0	0	0	0	0
7	Control	216	9.8	0	0	0	0	0	0	0
8	Control	219	9.4	0	0	0	0	0	0	0
9	Control	216	9.6	0	0	0	0	0	0	0
10	Control	213	9.8	0	0	0	0	0	0	0
1	MNU	203	9.8	0	4	6	0	1	0	0
2	MNU	222	11.5	0	1	4	0	2	0	0
3	MNU	230	11.3	4	1	0	0	0	0	0
4	MNU	204	12.0	2	4	0	0	0	0	0
5	MNU	217	8.6	4	0	0	0	0	0	0
6	MNU	213	9.1	2	3	1	0	0	0	0
7	MNU	203	9.7	0	3	2	0	0	0	0
8	MNU	235	11.7	3	1	0	0	0	0	0
1	MET	150	7.4	4	0	0	0	0	0	0
2	MET	220	10.0	4	1	0	0	0	0	0
3	MET	218	11.0	4	0	0	0	0	0	0
4	MET	216	11.6	4	0	0	0	0	0	0
5	MET	217	11.3	3	1	0	0	0	0	0
6	MET	230	9.6	4	0	0	0	0	0	0
7	MET	199	11.2	2	3	0	0	0	0	0
8	MET	240	8.9	4	0	0	0	0	0	0

Supplementary Table S3: The general features of the MNU-induced bladder cancer treated with or without MET at week 12

NO.	Group	Weight (g)	Glucose (mmol/L)	Hyperplasia/Dysplasia			Papillary	CIS	T1/T2	SCC
				Mild	Moderate	Severe				
1	Control	230	7.5	0	0	0	0	0	0	0
2	Control	230	7.6	0	0	0	0	0	0	0
3	Control	228	7.9	0	0	0	0	0	0	0
4	Control	223	7.6	0	0	0	0	0	0	0
5	Control	231	7.9	0	0	0	0	0	0	0
6	Control	239	8.0	0	0	0	0	0	0	0
7	Control	239	8.0	0	0	0	0	0	0	0
8	Control	242	7.9	0	0	0	0	0	0	0
9	Control	215	9.0	0	0	0	0	0	0	0
10	Control	234	8.1	0	0	0	0	0	0	0
1	MNU	265	5.8	0	0	0	2	1	0	0
2	MNU	230	6.5	0	0	0	2	0	4	2
3	MNU	263	6.4	0	0	0	3	0	1	0
4	MNU	252	7.5	0	0	0	2	0	0	2
5	MNU	250	7.0	0	0	0	2	0	4	3
6	MNU	290	4.9	0	0	0	0	0	4	2
7	MNU	240	6.3	0	0	0	3	0	0	0
8	MNU	198	5.7	0	0	0	1	1	3	0
9	MNU	220	6.1	0	0	0	2	0	4	7
10	MNU	231	9.0	0	0	0	0	0	1	0
11	MNU	228	11.3	0	0	0	0	1	2	1
12	MNU	198	8.3	0	0	0	0	0	3	1
13	MNU	181	5.7	0	0	0	0	0	2	0
14	MNU	216	14.5	0	0	0	2	0	0	0
1	MET	248	7.7	0	0	0	2	0	2	2
2	MET	256	7.4	0	0	0	3	0	0	0
3	MET	232	7.8	0	0	0	2	0	1	2
4	MET	270	7.8	0	0	0	1	0	2	3
5	MET	250	5.8	0	0	0	2	0	0	0
6	MET	235	5.9	0	0	0	2	0	2	0
7	MET	204	5.5	0	0	0	3	0	0	2
8	MET	190	4.4	0	0	0	0	0	4	6
9	MET	197	5.3	0	0	0	3	0	0	0

(*Continued*)

NO.	Group	Weight (g)	Glucose (mmol/L)	Hyperplasia/Dysplasia			Papillary	CIS	T1/T2	SCC
				Mild	Moderate	Severe				
10	MET	182	4.8	0	0	0	4	0	0	1
11	MET	200	6.8	0	0	0	2	0	0	0
12	MET	223	4.2	0	0	0	3	0	2	2
13	MET	200	4.6	0	0	0	4	0	2	2
14	MET	209	4.7	0	0	0	2	0	1	0
15	MET	206	9.6	0	0	0	3	0	2	1
16	MET	171	9.7	0	0	0	3	0	0	0
17	MET	212	10.8	0	0	0	4	0	1	0
18	MET	243	9.8	0	0	0	3	0	0	0
19	MET	197	11.4	0	0	0	4	0	0	0
20	MET	241	3.7	0	0	0	3	0	2	4

Supplementary Table S4: Immunohistochemical scores(IHS) of KI-67, OCT3/4 and P-stat3

Weeks	Group	IHS		
		KI-67	OCT3/4	P-stat3
Week 3	MNU	4	4	2
	MNU	3	4	2
	MNU	4	4	3
	MNU	2	2	2
	MET	2	2	1
	MET	1	2	2
	MET	2	1	1
	MET	1	1	1
Week 6	MNU	4	6	3
	MNU	8	6	6
	MNU	6	6	4
	MNU	6	4	4
	MET	4	4	2
	MET	3	4	3
	MET	4	2	2
	MET	4	2	2
Week 12	MNU	6	9	6
	MNU	9	6	9
	MNU	8	9	6
	MNU	6	6	8
	MET	4	2	4
	MET	4	3	4
	MET	6	6	4
	MET	4	3	6
